# Surgeons against Juries and Shysters

**Published:** 1870-02

**Authors:** 


### Miscellaneous.

### Surgeons Against Juries and Shysters.

The recent prosecution of Dr. A. D. Hall, by a patient, on charge of malpractice causing the loss of an eye, for the purpose of recovering damages, is an illustration of the liability of surgeons of hospitals to suffer outrageous imposition while scientifically and humanely performing their duty. The case and its issue will certainly impress surgeons with the danger to which they are daily exposed from vicious patients when led on by their cupidity or that of their friends or attorneys.

The plaintiff in this case, a young woman, applied to the Wills Ophthalmic Hospital, nearly two years ago, and was registered on the books as suffering with staphyloma of the cornea. The object of the operation, the removal of the anterior part of the eyeball, performed at her request, in the presence of a consultation of the surgeons, was to get rid of disfigurement and to enable her to wear an artificial eye.

The performance of the operation was also influenced by the existence of sympathetic irritation in the other eye.

The evidence of all with whom she came in contact in the Hospital proved that she fully comprehended the benefits to be gained by the operation, that she was well satisfied with the treatment she received, that she made inquiries in regard to the cost of an artificial eye, and that she continued to return as an out-patient after her discharge. These facts were verified by abundant evidence of attending surgeons, resident surgeon, nurses, and the records of the institution. Evidence might also have been produced that the woman was known to have been blind in the eye operated on for several years

Instead of being thankful for the great benefit secured by the partial removal of the sightless and unsightly eye, she is brought into a court room with the collapsed orbit to pitifully appeal, through the oratory of an ingenious attorney, for damages for the apparent loss!

The plaintiff's case rested upon the evidence of some very illiterate persons, that she was not entirely blind in the eye. It was admitted that the eye had been for three years partially covered by a "speck" or a "scum." The woman also admitted in the court room that she had never tested whether the eye was entirely blind, by closing the other eye. An "expert" was called forth who stated that he had practiced medicine "excepting when in the *oil business*," but was compelled to acknowledge, when questioned, that he knew nothing of the surgery of the eye, and he was at once dismissed by the judge.

The claim was for ten thousand dollars.

The defence was abundantly sustained by the evidence alluded to and also by prominent surgeons, called in as experts, including Doctors Gross, Pancoast, Lewis and Morton.

The jury rendered a verdict for the plaintiff for *eight hundred dollars*. We are gratified to know that this unjust verdict was instantly set aside by Judge Stroud, as against the evidence.

The absurdity of the verdict, as to the amount of damages awarded, is evident. Dr. Hall either did or did not culpably destroy vision in the eye. If he really had done what she attempted to prove, the amount asked for was not too much, and the smallness of the sum awarded makes it apparent that the jury could not have considered him answerable for the loss of the eye.

Medical practioners have repeatedly suffered from the ignorance and mistaken sympathy of a "jury of their peers," but it is gratifying in this case, as it has been in some other instances, that the intelligence of an upright judge has reversed the wrongful verdict.

Two other suits for malpractice, against most worthy members of the profession, are soon to be tried in this city and it is to be hoped that they may not be presented before juries made up of men who can see in a deformed limb, rescued from entire loss, or in a vacant orbit which has saved another eye from blindness, only blundering or thirst for blood, on the part of the humane and scientific practioner.—*Philadelphia Reporter.*

